# Supplementary material for: Thermodynamic driving forces in contact electrification between polymeric materials
Source: Nat Commun. 2024 Mar 23;15:2616. doi: 10.1038/s41467-024-46932-2 (PMC10960812; doi:10.1038/s41467-024-46932-2)
Supplement: Supplementary file 1 — Supplementary Information [file 41467_2024_46932_MOESM1_ESM.pdf]

Supplementary Information (SI) Appendix for

Thermodynamic driving forces in contact electrification between polymeric materials

Hang Zhang, Sanakaran Sundaresan, Michael A. Webb

Corresponding author: Michael A. Webb, mawebb@princeton.edu

This PDF file includes:

Supplementary Notes:

- Suppl. Note 1. Comparison to Triboelectric Matrices Sourced from Literature Data
- Suppl. Note 2. Comparison of Ion Distributions in Proximity to PVA
- Suppl. Note 3. Distance Analysis of Two-surface Free-energy Calculations
- Suppl. Note 4. Free-energy Calculations of Ion in The Free Water Droplet
- Suppl. Note 5. Additional Simulation Details
- Suppl. Note 6. Additional Description of Single-surface Free-energy Calculations
- Suppl. Note 7. Additional Description of Two-surface Free-energy Calculations

Supplementary Figures:

- Suppl. Fig. 1 to 6

Suppl. Note 1. Comparison to Triboelectric Matrices Sourced from Literature Data

Triboelectric series were collected from ten publications from 1898 to 2022 that featured at least a subset of the six polymers investigated in this work. The reported triboelectric series, which are a common representation of contact charging experiments, were then formulated as a matrix as introduced in the main text. Because many experiments utilize a "probe" material rather than direct material contact, we note that this matrix is an idealistic representation. Nevertheless, in the framework of thermodynamics, even if the charging were completely mediated by a third "probe" material, the equilibrium condition would reflect the same charging pattern as if the target pair had been in direct contact. Suppl. Fig. 1 summarizes all the data. The figure overall conveys some level of inconsistency in experimental settings even for the same reported materials; however, there are also some broadly conserved trends. For example, most of the top-right corner and bottom-left appear consistently colored for the experimental data, and these trends are further reflected in the predictions made by the free-energy calculations.

Suppl. Note 2. Comparison of Ion Distributions in Proximity to PVA

In Fig. 3A, the distribution of H_3O^+ and OH^- appear similar along the single dimension relative to the polymer interface. However, Suppl. Fig. 2 illustrates that the two ions do exhibit differences when resolving positioning relative to both the polymer interfaces and water interfaces. In Suppl. Fig. 2, positioning along the diagonal but away from the origin indicates residence resides toward the center of the droplet, as it is simultaneously distanced from both interfaces. Meanwhile, positioning in a lower horizontal band towards $y = 0$ suggests the ion is close to a water interface; however, it need not be close to a polymer interface (moving right), implying that the ion is closer to a water-vapor interface. Therefore, the simulations indicate that H_3O^+ displays some preference to reside near the water-vapor interface, while OH^- is always found in the interior of the water droplet. This behavior is generally preserved across all surfaces.

Suppl. Note 3. Distance Analysis of Two-surface Free-energy Calculations

To guide selection of distances for the two-surface free-energy calculations, a set of preliminary simulations at varying distances ($d = 15, 25, 40, 55$ Å) for PMMA-PVC pairings. PMMA-PVC were selected based on the predictions to acquire the most positive/negative charge from the single-surface thermodynamic integration calculations; the span of distances allowed for the formation of a water bridge between the two surfaces without any direct contact of PMMA and PVC atoms. Suppl. Fig. 3A provides the corresponding free-energy profiles as a function of ionic dipole. At the small separation of $d = 15$ Å, preference for either ion to specific surfaces is not clearly evident. At larger separations ($d = 25, 40, 55$ Å), relative affinities become statistically discernible, with all separations yielding qualitatively similar interpretations. Consequently, a first group of simulations amongst all surface pairs were performed with $d = 25$ Å (Suppl. Fig. 3). For a subset of pairs (N66-PE, N66-PTFE, N66-PVC, PE-PTFE, PMMA-

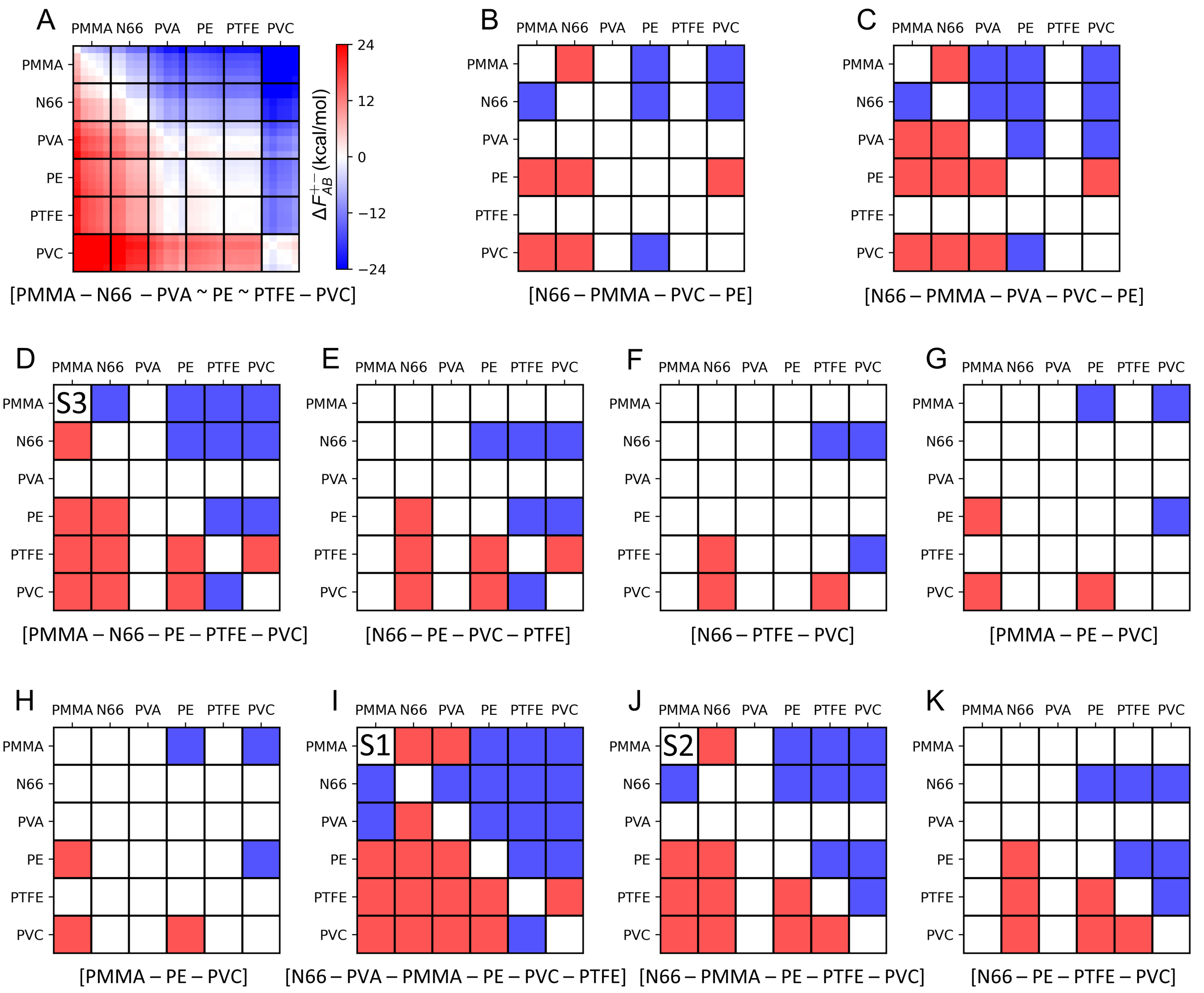

Supplementary Figure 1: **Different triboelectric matrices generated from previous published triboelectric series.** The order of six polymer surfaces is in the order of this work. The linear versions of the series are listed below the matrices. Three series S1, S2, and S3 are labeled in the top left corner of the matrices. For panel A, the color indicates the free-energy difference ΔF_{AB}^{+-} from the simulations. For other panel, the blue color indicates that the row surface is positively charged and the column surface is negatively charged. The red color indicates the opposite situation. The origins of these triboelectric series are as follows: (A) This work (B) Ref. 1 (C) Ref. 2 (D) Ref. 3 (E) Ref. 4 (F) Ref. 5 (G) Ref. 6 (H) Ref. 7 (I) Ref. 8(J) Ref. 9 (K) Ref. 10

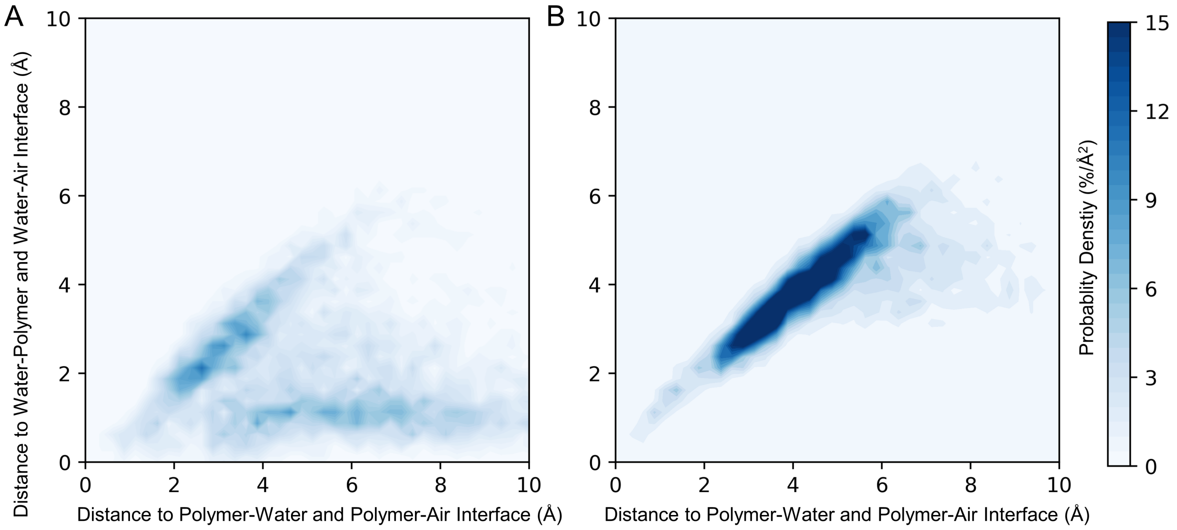

Supplementary Figure 2: **Comparison of ion distribution within water droplets on amorphous, atactic PVA.** (A) The probability density of H_3O^+ relative to interfaces involving polymer atoms (horizontal axis) and interfaces involving water atoms (vertical axis). (B) The same as (A) but for OH^-. While OH^- appears distributed purely along the diagonal, H_3O^+ possesses significant probability density at small distances to water interfaces.

PTFE, and PVC-PTFE), relative surface affinities were not obvious at $d = 25$ Å, and additional simulations were run at $d = 40$ Å. For PVA-PE and PVA-PTFE, simulations at larger d were not feasible due to the hydrophobicities of PE and PTFE relative to PVA (Suppl. Fig. 4), which resulted in all water residing on PVA and no stable water bridge. For all polymer pairs, a 10 ns equilibrium simulation was performed. The preliminary simulations used 7.5 ns of simulation in each biasing window. The first group of simulations were run for 15 ns for each biasing window. An additional 7.5 ns simulation are performed for $|p_z| < 10$ Å to get better sampling of the small $|p_z|$ region. For pairs that exhibited flatter free-energy profiles, an additional 7.5 ns of simulation were run to assess convergence. The second group of simulations were performed for 15 ns in each biasing window.

Suppl. Note 4. Free-energy Calculations of Ion in The Free Water Droplet

To further understand our single free-energy results, we performed calculations involving a free water droplet. The simulation systems are generated by placing a water molecules in a spherical geometry at the center of simulation cell; subsequent simulation procedures follow those of the single-surface free-energy calculations. Suppl. Fig. 5 shows that the free energy of adding an ion to a free water droplet are statistically indistinguishable from those of adding to droplets on PE and PTFE. This implies that the ions within free water droplets are stabilized to a similar extent as on hydrophobic surfaces. By extension, these results suggest that the free-energy

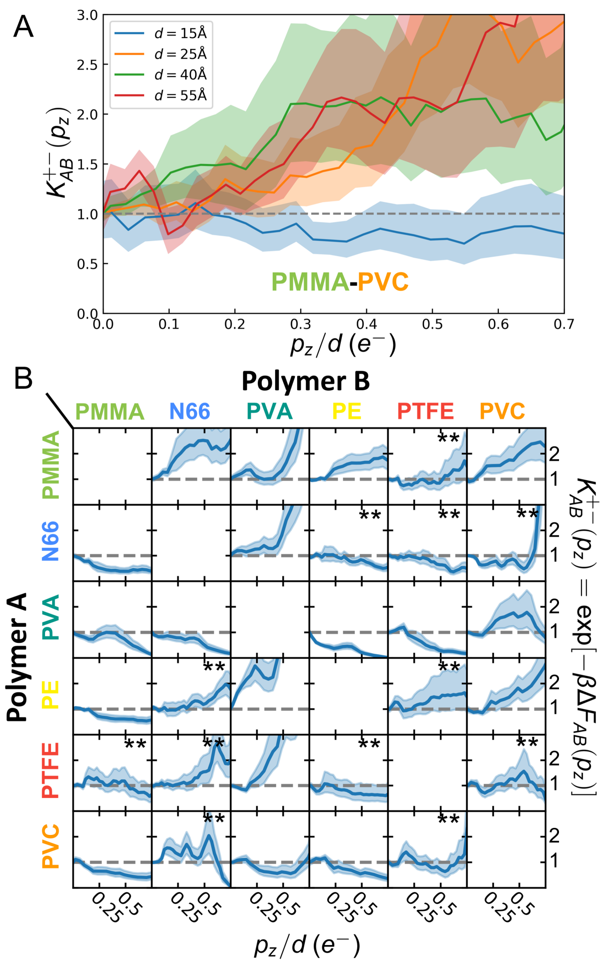

Supplementary Figure 3: **Dependence of relative ion-surface affinities on distance between surfaces.** (A) Comparison of relative ion partitioning between surfaces as a function of ionic dipole, $K^{+-}_{AB}(p_z)$, as surfaces are separated at distances of $d = 15, 25, 40$, and 55 Å. (B) Summary of all $K^{+-}_{AB}(p_z)$ for all pairs of surfaces separated by $d = 25$ Å. The surface pairs denoted with "**" are further simulated at $d = 40$ Å due to their relatively weak dependence on p_z; these results are shown in the main text. The shaded regions indicate statistical uncertainties that reflect the standard error of the mean as estimated from bootstrap resampling.

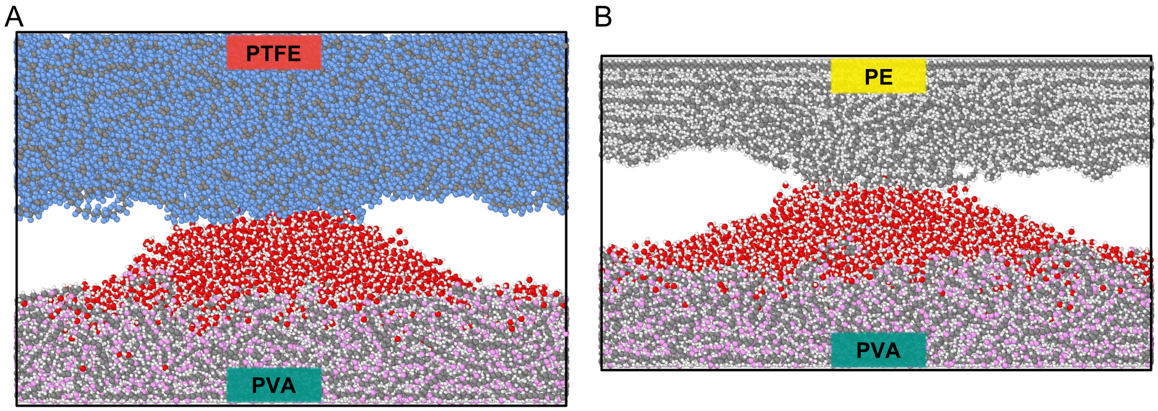

Supplementary Figure 4: **Effect of surface hydrophobicity on two-surface free-energy calculations.** (A) Configurational snapshot of water bridge forming between PTFE and PVA separated by $d = 25$ Å. (B) Configurational snapshot of water bridge forming between PE and PVA separated by $d = 25$ Å. Both PTFE and PE are substantially more hydrophobic than PVA. Consequently, the majority of water molecules in the system are recruited towards the PVA surface, which affects the accessible volume of ions. Both snapshots are rendered using OVITO (*11*). The atoms are colored such that carbon is gray, fluorine is green, hydrogen is white, oxygen in water and ion is red, and oxygen in PVA is pink.

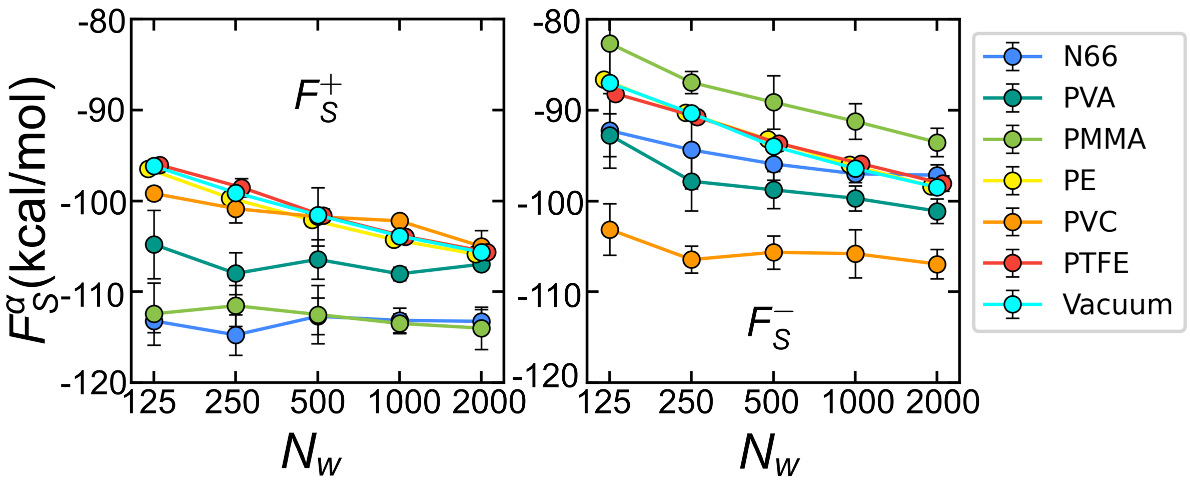

Supplementary Figure 5: **Results of free-energy calculations for a free water droplet and amorphous polymers.** The free water droplet results are colored by cyan and other polymers are colored as in the main text. The markers of PE and PTFE are shifted along x-axis to better display the results. Error bars reflect statistical uncertainties reported as the standard error of the mean calculated from independent thermodynamic integration trajectories.

trends of PE and PTFE are primarily induced by chemically specific effects moreso related to the other polymer surface in the contact-pair.

Suppl. Note 5. Additional Simulation Details

All MD simulations were conducted using version 3 Mar 2020 of the LAMMPS simulation package (*12*). The polymer-water systems were prepared using a methodology similar to our previous work (*13*), except that water-ions were also embedded at the center-of-mass of the water droplets as needed. Periodic boundary conditions were applied in the x and y dimensions, while the z dimension was extended with fixed boundaries featuring repulsive walls that generated forces in a direction perpendicular to the wall. Polymers were described with parameters obtained from the all-atom Optimized Potentials for Liquid Simulations (OPLS-AA) force field (*14, 15*), while water was described using the extended simple point charge model (SPC/E) (*16, 17*). The water ions were represented using a nonpolarizable force field that was parameterized to reproduce thermodynamic properties, such as solvation free energies in water (*18*). Real-space non-bonded interactions were truncated at 10 Å. Long-range electrostatics were handled using the particle-particle-particle-mesh Ewald summation method (*19*) with a convergence accuracy of 10^{-5}; this method was modified to accommodate the slab geometry with a non-periodic z dimension (*20*). Equations of motion were evolved using a velocity-Verlet integration scheme with a 1 fs timestep. A rigid geometry was maintained for all water and ion molecules using the SHAKE algorithm (*21*). Unless otherwise specified, temperature

was controlled at 300 K using a Nosé–Hoover thermostat (*22*) with a damping constant of 100 fs. Following system preparation, 20 ns equilibrium simulations were conducted. Subsequently, 20 ns production simulations were performed for all systems, and an additional 20 ns of simulations were conducted for $N_{\mathrm{w}} = 2000$ for structural analysis. Interfaces were identified according to the approach of Willard and Chandler; (*23*) these calculations were facilitated by the Pytim package (*24*) using the same settings as in our previous work (*13*).

Suppl. Note 6. Additional Description of Single-surface Free-energy Calculations

Thermodynamic Integration (TI) was used to compute the free energy of adding one ion to the water droplet. Prior equilibrated configurations were used as the initial configuration for simulations used for TI:

$$F_{+/-}(N_{\mathrm{w}}) = \int_0^1 \left\langle \frac{dU(\lambda,\vec{q})}{d\lambda} \right\rangle_\lambda d\lambda = \sum_{i=1}^{12} w_i \left\langle \frac{dU(\lambda,\vec{q})}{d\lambda} \right\rangle_{\lambda_i} . \tag{1}$$

In Eq. (1), $\langle \cdot \rangle_\lambda$ denotes an ensemble-average obtained using λ, which is the thermodynamic path variable such that $\lambda = 0$ corresponds to a state with only water droplet and the polymer surface and $\lambda = 1$ corresponds to the state with a water droplet, a water ion, and a polymer surface. As shown, the integral is numerically approximated using 12-point Gauss-Legendre quadrature with $\lambda \in \{0.00922, 0.04794, 0.11505, 0.20634, 0.31608, 0.43738, 0.56262, 0.68392, 0.79366, 0.88495, 0.95206, 0.99078\}$. For the configurational potential energy with λ , $U(\lambda, \vec{q})$, we utilized a soft-core potential (*25*) for pairwise Coulombic and Lennard-Jones potential energy contributions involving the ion molecule:

$$U_{\mathrm{LJ}}(r_{ij}, \sigma_{ij}, \varepsilon_{ij}, \lambda) = 4\lambda\varepsilon_{ij} \left\{ \frac{1}{\left[0.5(1-\lambda)^2 + \left(\frac{r_{ij}}{\sigma_{ij}}\right)^6\right]^2} - \frac{1}{0.5(1-\lambda)^2 + \left(\frac{r_{ij}}{\sigma_{ij}}\right)^6} \right\} \tag{2}$$

and

$$U_{\mathrm{coul}}(r_{ij}, q_i, q_j) = \lambda \frac{q_i q_j}{\left[10(1-\lambda)^2 + r_{ij}^2\right]^{1/2}} . \tag{3}$$

The utilization of soft-core potentials allows for Eq. (2) and Eq. (3) to possess the same λ, as opposed to performing TI in two stages (e.g., first handling U_{LJ} terms and then U_{coul} terms). Because the simulation box is heterogeneous, we find this preferable for sampling efficiency as utilizing only Lennard-Jones interactions in the absence electrostatics causes the ion to predominantly explore the vast "vapor" phase. We note that the free energy depends explicitly on N_{w} and also there exists a subtle finite-size effect based on our heterogeneous system construction. Detailed discussion of this finite-size effect can be found in our previous work; (*13*) however, the effect is inconsequential in the construction of our free-energy differences.

Suppl. Note 7. Additional Description of Two-surface Free-energy Calculations

Two-surface systems were prepared by flipping and adding one equilibrated polymer-water system with one ion type to another with the opposite ion. For each pair of polymers, one of three amorphous systems for one polymer was randomly chosen and paired with a randomly chosen system for the other polymer. The distances of two surfaces, d, was then set based on average surface interface position. The $F_{AB}(p_z)$ was calculated using umbrella sampling with statistical re-weighting via the weighted histogram analysis method (*26*). Data was collected across 36 windows that each employ a harmonic biasing potential on p_z. The biasing potentials utilize spring constants of 47.8011 kcal/mol and equilibrium positions at -35 to 35 Å in 2 Å increments. Sampling was facilitated using version 2.8.1 of PLUMED (*27*).

We note that the classical force field for H_3O^+ and OH^- was not parameterized (*18*) to handle possible recombination of ionic species into neutral water molecules. To focus sampling on configurations for which the ions are separate charged species and within the realm of applicability of the force field, we modified the non-bonded interaction between oxygen atoms on H_3O^+ and OH^- to be repulsive at distances less than approximately 4 Å. This is practically achieved by increasing ε in the Lennard-Jones potential to 1.0kcal/mol (Suppl. Fig. 6). The net effect of this modification is that ions do not form unphysical hydrogen bonds, which would otherwise arise using the original parameters. Consequently, $F_{AB}(p_z)$ conditionally depends on the ions being separate charged species that are separated by approximately 3 Å. Formally, $F_{AB}(p_z)$ is biased by this modified potential, but Suppl. Fig. 6 shows that this effectively negligible beyond 4 Å, and so re-weighting was not performed with respect to this bias.

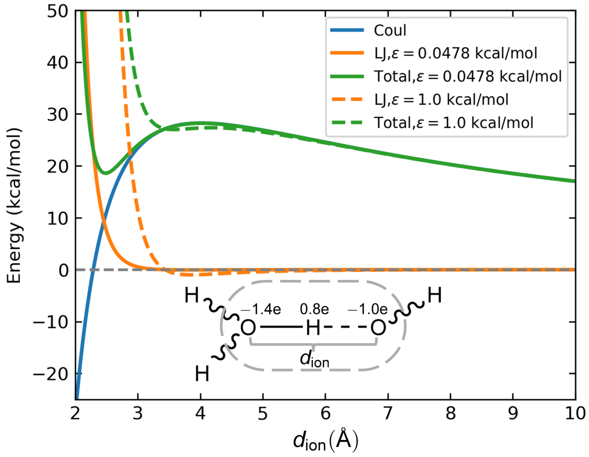

Supplementary Figure 6: **Comparison of pairwise H_3O^+-OH^- interaction energies with and without modified oxygen-oxygen interaction.** The original model of Ref. (*18*) was not parameterized to directly capture OH^--H_3O^+ interactions and does not address recombination into neutral species; conventional mixing rules results in an unphysical hydrogen bond between charged species at close separation distances. By increasing ε from 0.0478 kcal/mol to 1.0 kcal/mol, the H_3O^+ and OH^- are biased to maintain separation distances that avoid such behavior. Energies are computed as a function of distance between oxygen atoms d_{ion} while the angle formed between O(H_3O^+)-H(H_3O^+)-O(OH^-) is 180°. The inset schematic illustrates the specific geometry used.

Supplementary References

1. Coehn, A. Ueber ein gesetz der electricitätserregung. *Annalen der Physik* **300**, 217–232 (1898).

2. Hersh, S. & Montgomery, D. Static electrification of filaments. *Textile Research Journal* **25**, 279–295 (1955).

3. Henniker, J. Triboelectricity in polymers. *Nature* **196**, 474–474 (1962).

4. Adams, C. K. *Nature's electricity* (Tab Books, 1987).

5. Jonassen, N. *Electrostatics* (Springer US, 1998).

6. Iuga, A., Calin, L., Neamtu, V., Mihalcioiu, A. & Dascalescu, L. Tribocharging of plastics granulates in a fluidized bed device. *Journal of Electrostatics* **63**, 937–942 (2005).

7. Park, C. H., Park, J. K., Jeon, H. S. & Chun, B. C. Triboelectric series and charging properties of plastics using the designed vertical-reciprocation charger. *Journal of Electrostatics* **66**, 578–583 (2008).

8. McCarty, L. & Whitesides, G. Electrostatic charging due to separation of ions at interfaces: Contact electrification of ionic electrets. *Angewandte Chemie International Edition* **47**, 2188–2207 (2008).

9. Zou, H. *et al.* Quantifying the triboelectric series. *Nature Communications* **10**, 1427 (2019).

10. Shin, E.-C., Ko, J.-H., Lyeo, H.-K. & Kim, Y.-H. Derivation of a governing rule in triboelectric charging and series from thermoelectricity. *Physical Review Research* **4**, 023131 (2022).

11. Stukowski, A. Visualization and analysis of atomistic simulation data with OVITO–the open visualization tool. *Modelling and Simulation in Materials Science and Engineering* **18**, 015012 (2009).

12. Thompson, A. P. *et al.* LAMMPS - a flexible simulation tool for particle-based materials modeling at the atomic, meso, and continuum scales. *Computer Physics Communications* **271**, 108171 (2022).

13. Zhang, H., Sundaresan, S. & Webb, M. A. Molecular dynamics investigation of nanoscale hydrophobicity of polymer surfaces: What makes water wet? *The Journal of Physical Chemistry B* **127**, 5115–5127 (2023).

14. Jorgensen, W. L., Maxwell, D. S. & Tirado-Rives, J. Development and testing of the OPLS all-atom force field on conformational energetics and properties of organic liquids. *Journal of the American Chemical Society* **118**, 11225–11236 (1996).

15. Siu, S. W., Pluhackova, K. & Böckmann, R. A. Optimization of the OPLS-AA force field for long hydrocarbons. *Journal of Chemical Theory and Computation* **8**, 1459–1470 (2012).

16. Berendsen, H., Grigera, J. & Straatsma, T. The missing term in effective pair potentials. *Journal of Physical Chemistry* **91**, 6269–6271 (1987).

17. Chatterjee, S., Debenedetti, P. G., Stillinger, F. H. & Lynden-Bell, R. M. A computational investigation of thermodynamics, structure, dynamics and solvation behavior in modified water models. *The Journal of Chemical Physics* **128**, 124511 (2008).

18. Bonthuis, D. J., Mamatkulov, S. I. & Netz, R. R. Optimization of classical nonpolarizable force fields for OH^- and H_3O^+. *The Journal of Chemical Physics* **144**, 104503 (2016).

19. Hockney, R. W. *Computer simulation using particles* (A. Hilger, 1988).

20. Yeh, I. C. & Berkowitz, M. L. Ewald summation for systems with slab geometry. *Journal of Chemical Physics* **111**, 3155–3162 (1999).

21. Ryckaert, J.-P., Ciccotti, G. & Berendsen, H. J. Numerical integration of the cartesian equations of motion of a system with constraints: molecular dynamics of n-alkanes. *Journal of computational physics* **23**, 327–341 (1977).

22. Hoover, W. G. Canonical dynamics: Equilibrium phase-space distributions. *Physical Review A* **31**, 1695–1697 (1985).

23. Willard, A. P. & Chandler, D. Instantaneous liquid interfaces. *The Journal of Physical Chemistry B* **114**, 1954–1958 (2010).

24. Sega, M., Hantal, G., Fábián, B. & Jedlovszky, P. Pytim: A python package for the interfacial analysis of molecular simulations (2018).

25. Beutler, T. C., Mark, A. E., van Schaik, R. C., Gerber, P. R. & Van Gunsteren, W. F. Avoiding singularities and numerical instabilities in free energy calculations based on molecular simulations. *Chemical physics letters* **222**, 529–539 (1994).

26. Kumar, S., Rosenberg, J. M., Bouzida, D., Swendsen, R. H. & Kollman, P. A. THE weighted histogram analysis method for free-energy calculations on biomolecules. i. the method. *Journal of Computational Chemistry* **13**, 1011–1021 (1992).

27. Tribello, G. A., Bonomi, M., Branduardi, D., Camilloni, C. & Bussi, G. Plumed 2: New feathers for an old bird. *Computer physics communications* **185**, 604–613 (2014).
