# Supplementary material for: Thermodynamic driving forces in contact electrification between polymeric materials
Source: Nat Commun. 2024 Mar 23;15:2616. doi: 10.1038/s41467-024-46932-2 (PMC10960812; doi:10.1038/s41467-024-46932-2)
Supplement: Supplementary file 3 — Description of Additional Supplementary Files [file 41467_2024_46932_MOESM3_ESM.pdf]

Description of Additional Supplementary Files:

Supplementary Data 1: Summary of results from thermodynamic integration of ion addition to water droplets atop polymer surfaces.

Supplementary Data 2: Summary of results from umbrella sampling on the ionic dipole within water channels formed between two polymer surfaces.

Supplementary Data 3: Simulation configuration data files. Initial and final configurations are provided for each surface in LAMMPS data file format.
